# Burden of and risk factors for sexual violence among women with and without disabilities in two sub-Saharan African countries

**DOI:** 10.1080/16549716.2022.2077904

**Published:** 2022-07-01

**Authors:** Pierre De Beaudrap, Charles Mouté, Estelle Pasquier, Alice Tchoumkeu, Carole Dongmo Temgoua, Aida Zerbo, Muriel Mac-Seing, Gervais Beninguisse

**Affiliations:** a CEPED, Institut de Recherche pour le Développement, Université Paris Cité, INSERM, Paris, France; b Direction de la Recherche, de la Coopération et de l'Appui Technique, Institut de Formation et de Recherche Démographique (IFORD), Yaoundé, Cameroon; c Bureau Central des Recensements et des Etudes de Population (BUCREP), Yaoundé, Cameroon; d Health Department, Initiative HIV/AIDS, Tuberculosis, Malaria, Paris, France; e Department of Public Health and Primary Care, Ghent University, Gent, Belgium; f Department of Public Health, Institute of Tropical Medicine, Antwerp, Belgium; g Centre for Global Health, Dalla Lana School of Public Health, University of Toronto, Toronto, Ontario, Canada

**Keywords:** Disability, gender-based violence, Cameroon, Burundi, social epidemiology

## Abstract

**Background:**

Available data suggest that women with disabilities have an increased risk of sexual violence, but little is known about the situation of those women living in resource-limited settings.

**Objectives:**

To assess the burden and examine the drivers of sexual violence among women with disabilities.

**Methods:**

This is a pooled analysis of two population-based surveys conducted in Cameroon and Burundi. Adults with and without disabilities were randomly recruited from the general population. Structured interviews were conducted at both sites to collect data on participants’ functional limitations, life-course history of sexual violence, education, employment, and resources. Only women with disabilities whose impairments started before the age of 10 years (n = 359) and women without disabilities (n = 720) are included in this analysis. The age-adjusted prevalence of violence was computed, and risk factors were assessed using a discrete survival regression and mediation analysis.

**Results:**

At both sites, the participants with disabilities had a lower education level and had an increased risk of food insecurity. The pooled age-adjusted prevalence of lifetime sexual violence was 19.8% (95%CI:15.3–24.3) among women with disabilities and 11.7% (95%CI:9.3–14.1) among those without disabilities (OR_ap_: 2.0, 95%CI:1.4–2.8). Women with cognitive limitations and those with visual impairments had the highest risk of sexual violence (OR_ap_: 3.5 (95%CI:2.0–6.3) and 2.7 (95%CI:1.4–5.0), respectively). Over the life course, the risk of sexual violence was especially high among women with disabilities who had lived with an intimate partner before the age of 25 years (*p* < 0.001). Education level mediated approximately one-third of the total association between disability and sexual violence (*p* = 0.001). There was no evidence of an indirect effect through food insecurity.

**Conclusion:**

This study provides evidence of the high burden of sexual violence among women with disabilities who live in urban African contexts. The social environment and access to education may be key contributors to this vulnerability.

## Background

Violence against women is a major global concern that affects over one billion people around the world [1,2]. Although it has received increasing attention in recent years, there remains much to do to better understand the factors associated with violence against women and ultimately improve prevention efforts. Some groups are particularly affected, due to being more exposed to violence and/or less likely to protect themselves or able to find help. Women with disabilities constitute one of the vulnerable groups and have received limited attention in the literature.

The Global Survey on Violence against Women released in 2013, which provided key figures on the prevalence of sexual and intimate partner violence, did not specifically examine the population with disabilities [3]. The World Report on Disability, which, to date, represents the most comprehensive report on the global situation of people with disabilities, contains only a brief section on violence against people with disabilities [4]. A systematic review accompanied by a meta-analysis on the frequency of violence against people with disabilities was published at the same time and showed that people with disabilities are significantly more often victims of violence than those without disabilities [5]. However, the conclusion of this analysis underscores the lack of data on certain types of violence (particularly sexual violence), on people with certain types of disability, and on the situation in resource-limited countries. More recently, the prevalence of intimate partner violence (IPV) was compared between women with and women without disabilities using baseline data of 8,549 women (17% with disabilities) from seven violence-prevention programmes conducted in resource-limited countries [6]. The analysis showed that the risk of sexual IPV over the 12 months preceding the survey was twice as high among women with disabilities as it was among those without disabilities, and that association between disability and IPV was consistent across the different settings. Study participants were considered to have a disability if they reported severe functional limitations. However, as information on the timing of the disability was not available, it was not possible to disentangle whether the disability was a consequence of IPV or the reverse. In addition, the influence of social and economic factors on the risk of violence against women with disabilities were not analysed.

An increasing body of evidence shows that people with disability are at a higher risk of multidimensional poverty, which includes a lack of education, a lack of access to health services and employment and other forms of social exclusion [7]. Poverty has also been consistently identified as a risk factor for violence against women. For instance, education influences the risk of sexual violence by changing women’s acceptance of partner violence [8]. Women with low education achievement are therefore less likely to call into question norms based on masculine dominance. They are also more likely to have poorer employment outcomes later in life and are thereby more dependent on their partners. Economic poverty can also be a direct driver of violence. In more deprived households, poverty may result in a higher level of stress, anxiety, mental illness, and, eventually, a lack of emotional control. Social exchange theory asserts that men who have fewer resources may use violence as a means to increase power and control over their partner [9]. Accordingly, poverty is a potential mediating factor for the risk of sexual violence against women with disabilities. However, other pathways could be involved. For instance, the frequent social devaluation of women with disabilities reduces their ability to negotiate masculine dominance. They are also more vulnerable to predatory partners who seek to assert control over them [10]. At the community level, reduced social integration can result in increased dependence on the family and less opportunity to escape an abusive situation [11].

In this research, we address the following three questions: 1) What is the burden of sexual violence among women who grew up with a disability? 2) Is there a specific subgroup or life period in which there is a higher risk of sexual violence? 3) To what extent do social and economic inequalities contribute to the vulnerability of women with disabilities to sexual violence?

## Methods

### Study design and participants

For this study we used data collected through two cross-sectional studies conducted in Yaoundé, Cameroon between 2 October 2014 and 30 November 2015 (the HandiVIH study [12]) and in Bujumbura, Burundi between 20 December 2017 and 20 December 2018 (the HandiSSR study [13]). These surveys were part of a series of studies conducted on the burden of HIV infection among people with disabilities in sub-Saharan Africa. The study sites were selected to reflect the important diversity of the epidemiological and social situations that may be encountered in sub-Saharan Africa. In both studies, a multistage sampling strategy was used to randomly select people with disabilities and matched controls from households in the general population. In the first stage, enumeration areas available from previous national surveys were sampled using probability proportional to the number of households. Each sampled enumeration area was enumerated again in an exhaustive way to update the data. Then, 100 households were randomly sampled in each sampled enumeration area from the updated list of households and contacted for the second stage. During this stage, study interviewers collected general information on the households and used the same Washington Group Disability Short Set (WGSS) questionnaire with each household’s member to ascertain the presence of activity limitation and to thereby identify people with disabilities eligible for the study [14].

All people aged 15 to 49 years with severe difficulties in at least one domain or with some difficulties in at least two domains of the WGSS questionnaire for ≥12 months were considered as living with disabilities and being eligible for the study [14]. When several persons with disabilities were identified within a single household, only one of them was recruited for the study. The WGSS covers the six functional domains of seeing, hearing, walking, cognition, self-care, and communication and was used in both studies. See supplementary material. For each person with a disability included in the study, a control person of a similar age and sex who lived in a different household in the same enumeration area and who did not meet the functional limitation criteria was recruited.

### Procedures

In both studies, face-to-face structured interviews were conducted at the home of theeligible participants to collect data on their activity limitations and difficulties in socialparticipation as well as on their life-course history of education, employment, resources(including reported difficulties regarding basic needs such as obtaining food), sexualviolence, sexual partnership, fertility using the life-grid method [15,16].

The questions used to measure the experience of sexual violence were as follows: (1) Have you ever been touched or caressed against your will by a stranger, a relative or an older person? and (2) Have you ever been forced to have sex when you did not want to? If the response to the second question was ‘yes’, then the respondent was asked to indicate when such experience occurred by using the life-grid. In addition, participants were asked to assess their experience at their first sexual intercourse, and an available item included ‘My partner forced me to have sex’. An additional question was included in the Burundi survey (designed after the survey in Cameroon) to measure IPV. For each intimate relationship ≥12 months, the participant was asked if he/she ever felt threatened by his/her partner or experienced physical or sexual violence. The wording of the different questions was carefully discussed with the teams and field tested. Moreover, pictograms and other communication tools (such as dolls, drawings and images) were used to facilitate communication between the study interviewers and respondents.

Details of the survey methods and procedures have already been described in detail elsewhere [13,17].

### Study population

In this analysis, the study population with disabilities was restricted to the participants for whom limitations occurred before the age of 10 years to ensure, as much as possible, that there was a chronological sequencing of disability and sexual violence. All participants without disabilities were included.

### Statistical analysis

The analysis was conducted in three steps. In the first step, a cross-sectional approach was adopted to estimate the overall burden of sexual and physical violence among women with and without disabilities. The age-standardized prevalence of lifetime sexual violence was computed separately for each site and disability status using direct standardization. Pooled estimations were performed with the generalized additive model (gam) to account for a nonlinear relation between the prevalence of sexual violence and age. Heterogeneity in the association found between the study sites was investigated by testing the significance of a quantitative interaction between the primary factors examined (e.g. disability) and the indicator of the study. Pooling was not performed when evidence of a change in the direction of the association between exposure (disability) and outcomes (sexual violence) between the studies was found. For instance, a positive association in one study and a negative one in the other. Subgroup analyses were performed to examine whether associations between the occurrence of sexual violence and disability varied according to the nature of the main activity limitation(s) (physical/visual/hearing/cognitive limitations).

In the second step, the risk of sexual violence across the women’s life course was examined using the data collected with a standardized biographic interview [16,18]. During this interview, the participants were asked to report on a calendar instrument for each year from age 10 to their current age at the time of the interview with whom they were living, what their main activities were, and whether they experienced food insecurity. The participants who were victims of sexual violence were asked to provide a chronology of this event by using the calendar instrument. The participants who did not report violence were considered censored at the date of interview. The hazard of sexual violence was estimated using a gam model with a log link and a smoothing component (splines) to represent the changes with age [19]. A discrete survival regression was used to assess the risk of sexual violence among the women with and without disabilities according to the following life-course variables [20]: 1) the type of activity (student, paid work, unpaid or informal work, housework, and no activity) and 2) the life environment, i.e. people with whom the participant lived (parents, extended family, intimate partner, and others). The ages of initial sexual violence were not available for eleven participants and were imputed using a parametric model for interval censored data. The final estimates adjusted for the additional imputation variability were computed using Rubin’s Rules [21].

In the last step, we examined whether multidimensional poverty could be a potential mediator of vulnerability to sexual violence associated with disability. The trajectories of two indicators of multidimensional poverty, specifically, education and food insecurity, were selected for this analysis because they provide complementary insights into the complex relation between multidimensional poverty and sexual violence. In resource-limited contexts where a large part of economic life is informal [22], food insecurity is a frequently used indicator to delineate the poverty line. There is good evidence of an association between food insecurity and different forms of violence [23,24]. Education is an important risk factor of sexual violence. In addition, it is considered a basic capability [25] and is included in many poverty measurements (e.g. the Human Development Index [26]). In this analysis, the following aspects of the education trajectories were considered one at a time: highest level achieved; early dropout from school (leaving school ≤10 years old), and current student status. The second group of indicators relates to the experience of food insecurity over the lifetime and includes the current level and the persistent experience of food insecurity. Food insecurity was defined based on self-reported difficulties of obtaining food on a two-level scale (high level versus no or mild concern about obtaining food). Food insecurity was deemed to be persistent when there was no period without reported food insecurity over the life course. The strength of the association between disability and sexual violence was estimated after adjusting for each of the potential mediating factors listed above, compared with the estimate of a model without these factors. Then, a mediation analysis was performed using the potential outcome framework with a discrete time survival analysis to assess the extent to which the association between disability and sexual violence was mediated by each of the aforementioned factors [27,28]. After a careful examination of the assumed causal pathway (Supplementary Figure), we identified that poverty in childhood could be a confounding factor between disability and poverty in adulthood. Therefore, all regressions included in the mediation analysis were adjusted for childhood poverty by using reported food insecurity at age 10 years as a proxy.

A detailed summary of the variables used in the different analyses is provided in the Supplementary Table. All analyses were performed using R [29].

### Ethics

The HandiVIH study was approved by the ‘Comité d’Ethique pour la Recherche en Santé Humaine’ in Cameroon (2014/03/431/L/CNERSH/SP), and the HandiSSR study was approved by the ‘Comité National d’Ethique pour la Protection des Êtres Humains participants à la Recherche Biomédicale et Comportementale’ in Burundi (visa n°214/CAB/SN/243/2017). Both studies were also approved by the ‘Comité Consultatif de Déontologie et d’Ethique’ of the Institut de Recherche pour le Développement (IRD).

## Results

### Study population

Of the 727 women with disabilities enrolled in the two studies (423 in Cameroon and 304 in Burundi), 359 reported that their activity limitations started before the age of 10 years and were therefore included in this analysis (Cameroon: 212, Burundi: 147). All women without a disability in the two studies were included (Cameroon: 423, Burundi: 297). The most frequent limitations observed in both studies were related to mobility difficulties (Table 1). There were significant differences between the two study sites in the proportion of women reporting visual difficulties (*p* = 0.03) and cognitive difficulties (*p* = 0.006).

**Table 1.** Characteristics of study participants.

	Cameroon			Burundi			Pooled age-adjusted OR	Hetero-geneity (p-value)
	Women with disability	Women without disability	OR_a_	Women with disability	Women without disability	OR_a_		
Age (year)								
15–25	94 (44)	143 (34)	ref	76 (52)	94 (31.5)	ref	ref	0.3
26–35	77 (36)	141 (33)	0.8 (0.6–1.2)	37 (25)	91 (30.5)	0.5 (0.3–0.8)	0.7 (0.5–0.9)	
36–45	41 (19)	139 (33)	0.5 (0.3–0.7)	34 (23)	112 (88)	0.4 (0.2–0.6)	0.4 (0.3–0.6)	
Education^#^								
<Primary	71 (41)	29 (9)	ref	53 (45)	45 (19)	ref	ref	0.01
Primary	36 (21)	92 (28)	0.2 (0.1–0.3)	37 (32)	58 (25)	0.5 (0.3–1.0)	0.3 (0.3–0.4)	
Secondary	55 (32)	145 (45)	0.2 (0.1–0.3)	23 (20)	108 (46)	0.2 (0.1–0.3)	0.2 (0.1–0.3)	
Higher education	10 (6)	59 (18)	0.1 (0–0.2)	4 (3)	23 (10)	0.2 (0.1–0.5)	0.1 (0.6–1.1)	
Household welfare score								
Q1	123 (29)	171 (21)	ref	119 (39)	249 (41)	ref	ref	0.3
Q2 to Q4	297 (71)	636 (79)	1.5 (1.0–2.1)	183 (61)	360 (59)	1.1 (0.7–1.6)	1.3 (1.0–1.7)	
Food insecurity ≤10 years								
Important concern	34 (8)	32 (4)	ref	42 (14)	35 (6)	ref	ref	0.9
Not/mild concern	380 (92)	774 (96)	2.2 (1.1–4.4)	260 (86)	574 (94)	2.1 (1.1–4.0)	2.1 (1.3–3.4)	
Food insecurity >15 years								
Important concern	73 (54)	111 (33)	ref	26 (29)	159 (68)	ref	ref	0.05
Not/mild concern	63 (46)	225 (67)	2.4 (1.6–3.5)	65 (71)	75 (32)	1.2 (0.7–2)	1.8 (0.6–1.1)	
Fosterage before age 10								
yes	85 (40)	145 (34)	ref	60 (41)	92 (31)	ref	ref	0.5
no	127 (60)	277 (66)	1.3 (0.9–1.8)	87 (59)	205 (69)	1.5 (1–2.3)	1.4 (1.1–1.8)	
Social support network								
≤1 person	71 (34)	74 (18)	ref	62 (42)	70 (24)	ref	ref	1
>1 persons	140 (66)	348 (82)	0.4 (0.3–0.6)	85 (58)	227 (76)	0.4 (0.3–0.7)	0.4 (0.3–0.6)	
Barriers to access basic services								
No major difficulty	15 (7)	101 (24)	ref	13 (9)	159 (54)	ref	ref	0.01
Major difficulties in ≥1 area	197 (93)	321 (76)	4.1 (2.3–7.3)	134 (91)	138 (46)	11.9 (6.4–21.9)	7.3 (4.8–11.2)	
Discussion about sexuality with parents								
Yes	41 (19)	81 (19)	ref	26 (21)	56 (19)	ref	ref	0.8
No	171 (81)	342 (81)	1.0 (0.7–1.5)	100 (79)	240 (81)	1.1 (0.7–1.9)	1.1 (0.8–1.5)	
Functional limitation								
Moving	85 (40)	-	-	52 (35)	-	-	-	0.4
Visual	50 (24)	-	-	20 (14)	-	-	-	0.03
Hearing	39 (18)	-	-	33 (22)	-	-	-	0.4
Cognitive	42 (20)	-	-	49 (33)	-	-	-	0.006

OR Odds Ratio; OR_a_ age-standardized odds ratio.

In Burundi, as in Cameroon, the participants with disabilities were at an increased risk of experiencing multidimensional poverty, which included a lower education level, an increased rate of food insecurity and a lower household wealth index (Table 1). These participants were also more likely to have been raised by persons other than their biological parents before age 10 years, to have a smaller support network, and to report difficulties related to the attitudes of people.

### Burden and risk of overall violence

The pooled age-adjusted prevalence of physical violence after age 15 years was 15.5% (95% confidence interval [95%CI] 11–20.1) among the women with disabilities and 13% (95%CI 9.8–16.1) among those without disabilities (*p* = 0.2). As displayed in Table 2, more participants reported physical violence in Burundi than in Cameroon (age-adjusted odds ratio [OR_a_]: 3.75, 95%CI 2.58–5.44). Questions on IPV and recent experiences of violence (<12 months) were included only in the study in Burundi, and it was found that the participants with disabilities were more likely to report recent physical violence than those without disabilities (13% versus 3.7%, respectively, p < 0.001) and more likely to report IPV when engaged in a long-term intimate relationship (17.1% versus 9.1%, respectively, *p* = 0.08).

**Table 2.** Age-standardized prevalence of violence and abuse among women with and without disability in Yaoundé (Cameroon) and Bujumbura (Burundi).

	Women with disability		Women without disability	
	Cameroon	Burundi	Cameroon	Burundi
Physical Violence				
In childhood	31.7 (24.4–38.9)	58.2 (50.4–66)	29.6 (24.4–34.8)	68.7 (60.4–77)
After 15 years	9.7 (5.2–14.3)	23.8 (15.1–32.5)	6.2 (3.7–8.7)	22.6 (16.2–28.9)
Recent (last 12 months)	NA	13 (6.8–19.2)	NA	3.7 (1.6–5.8)
Unwanted touching	NA	35.3 (25.7–44.9)	NA	36.8 (28.7–45)
Intimate partner violence^a^	NA	17.1 (7.7–26.5)	NA	9.1 (5.2–12.9)
Sexual violence	22 (16–28)	16.6 (10.2–23)	14.9 (11.4–18.3)	7.1 (4.1–10)

NA: not available in this survey; a: proportion estimated among the women engaged in an intimate relationship for at least 12 months.

### Burden and risk of sexual violence

The pooled age-adjusted prevalence of lifetime sexual violence was 19.8% (95%CI 15.3–24.3) among the women with disabilities and 11.7% (95%CI 9.3–14.1) among those without disabilities (Table 2). This translated into a pooled age-adjusted odds ratio [OR_ap_] of 2.0 (95%CI 1.4–2.8) (p-value for between sites heterogeneity: 0.2).

### Risk of sexual violence by type of limitation

**Figure 1.**
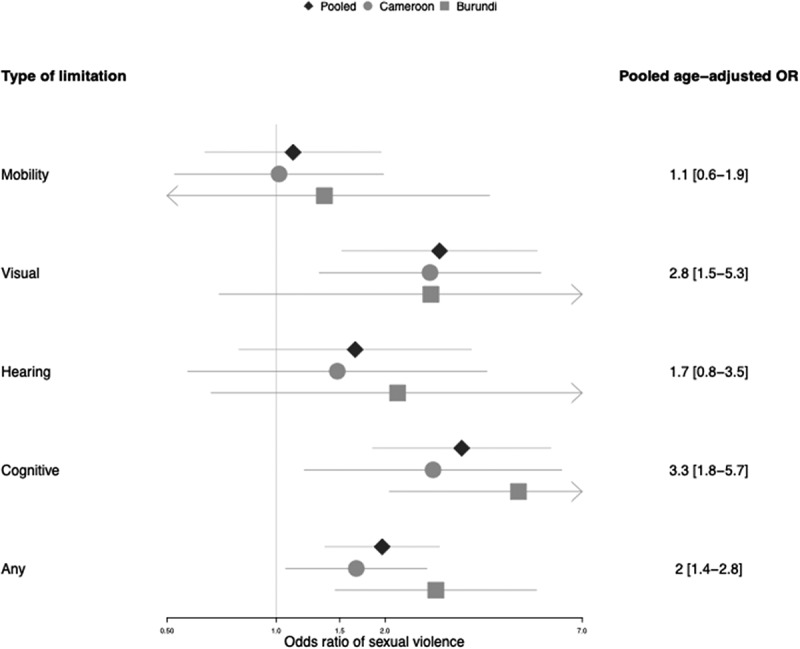
Age-adjusted lifetime sexual violence of women with disabilities compared to those without disabilities by types of limitation (mobility, visual, hearing and cognitive).

There was important heterogeneity in the risk of sexual violence across the different types of limitations (Figure 1). The women with cognitive limitations and visual limitations experienced the highest rates of sexual violence (OR_ap_: 3.5 [95%CI 2.0–6.3] and 2.7 [95%CI 1.4–5.0], respectively), while those with mobility limitations had a similar risk as the women without disabilities (OR_ap_ 1.1, 95%CI 0.6–1.9). There was no evidence of heterogeneity between the sites in the risk of sexual violence by the type of limitation (all p-values ≥ 0.4).

### Risk of sexual violence in women with and without disabilities from age 10 to 25 years

**Figure 2.**
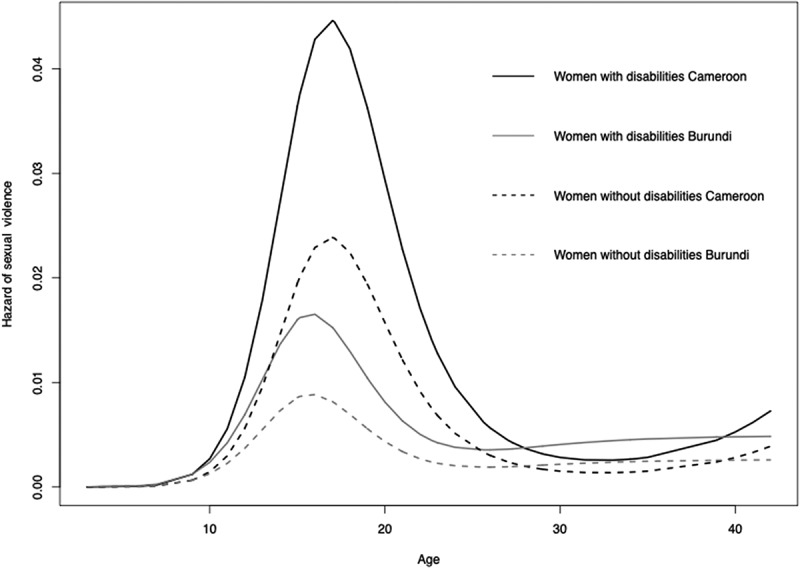
Estimated hazard of sexual violence among women with and without disabilities at the Burundi and Cameroon study sites.

As shown in Figure 2, the risk of sexual violence among the women with and without disabilities was greatest at the beginning of adulthood, although the magnitude and shape of the risk dynamic across age differed between the two study sites (*p* = 0.002).

In the remainder of this analysis of risk across the life course, we focus on the period from age 10 to 25 years to better understand how the association between sexual violence and disability varies with regard to the living environment during this critical period. Regarding their immediate social environment (i.e. with whom they were living), the women (with and without disabilities) who lived with an intimate partner were at an increased risk of sexual violence compared to those who lived with their parents (Table 3). Moreover, the risk of sexual violence associated with disability and living an intimate partner together was greater than the sum of the risks associated with each factor separately, which indicates a positive interaction on the additive scale (*p* = 0.08). Indeed, the joint age-adjusted pooled odds ratio of sexual violence associated with disability and living with an intimate partner was 23, 95%CI 9–58.5 while the odds ratio associated with disability alone was 2.8, 95%CI 1.6–4.9 and the odds ratio associated with living with an intimate partner alone 2.0, 95%CI 1.1–7.7.

**Table 3.** Pooled age-adjusted OR for sexual violence associated with disability by living environment between age 10 and 25.

	Disability		Interaction	
	Yes	No	Additive RERI (p-value)	Multiplicative OR (95%CI)
**Immediate social environment**				
Living with parents	ref	2.1 (1.3–3.5)		p = 0.05
Extended family	1.6 (0.9–2.8)	2 (1.1–3.7)	−0.7 (p = 0.2)	0.6 (0.3–1.3)
Living with a partner	1.8 (0.8–4.1)	8.9 (3.7–21.4)	8.2 (p = 0.08)	2.6 (0.9–7.9)
**Main activity**				
Student	ref	1.6 (1.0–2.7)		p = 0.9
Paid work	0.4 (0.1–3.3)	2.2 (0.5–9.2)	1.1 (p = 0.3)	3.0 (0.3–36)
Informal work	1.1 (0.4–2.8)	2.2 (0.9–5.6)	0.6 (p = 0.4)	1.3 (0.4–4.5)
No activity	0.8 (0.2–3.4)	2.2 (1.3–3.9)	0.9 (p = 0.09)	2.0 (0.4–9.5)
Homework	2.3 (0.7–7.8)	3.8 (0.9–15.9)	0.9 (p = 0.5)	1.1 (0.2–6.2)

The women without disabilities who lived with extended family were at increased risk of sexual violence compared to their peers who lived with their parents (OR_ap_ 2.7, 95%CI 1.6–4.6) but this association was not found among the women with disabilities (1.1, 95%CI 0.6–1.9).

Regarding the type of activity, the risk of sexual violence was similar among the women who were studying or working and women without reported activity (Table 3). The risk was higher in the participants with or without disabilities who were engaged in housework compared to students with and without disabilities. The OR_ap_ were 2.4, 95%CI 0.6–10.6 and 2.7, 95%CI 1.1–6.8, respectively.

### Socioeconomic factors associated with sexual violence and disability

Table 4 shows the main results of the mediation analysis. Approximately one-third of the total association between disability and sexual violence was mediated through the highest level of education achieved (*p* = 0.001). In contrast, current student status and early school drop-out did not mediate the risk of sexual violence associated with disability.

**Table 4.** Results of the mediation analysis. Pooled OR of sexual violence for women with disability adjusted for the different aspects of participants’ education or food insecurity trajectories and age (95%CI), and estimated proportion of the association mediated by the factor (p-value).

	All participants with disability		Cognitive limitation excluded		Only participants with cognitive limitation	
	OR_ap_ adjusted for factor	% mediated (p)	OR_ap_ adjusted for factor	% mediated (p)	OR_ap_ adjusted for factor	% mediated (p)
None (reference)	1.82 (1.30–2.54)	_	1.50 (1.03–2.2)	_	2.87 (1.77–4.64)	_
Education						
Highest level achieved	1.47 (1.03–2.09)	34% (<0.001)	1.35 (0.91–1.99)	27% (0.06)	1.71 (0.96–3.07)	49% (<0.001)
Early school drop-out	1.71 (1.21–2.42)	10% (0.2)	1.50 (1.02–2.20)	<1% (0.9)	2.66 (1.49–4.76)	7% (0.7)
Cumulative time spent studying	1.68 (1.18–2.38)	13% (0.08)	1.48 (1.01–2.18)	<1% (0.8)	2.54 (1.42–4.54)	11% (0.5)
Current student status	1.78 (1.27–2.51)	3% (0.3)	1.50 (1.02–2.20)	<1% (0.96)	2.92 (1.73–4.93)	<1% (0.8)
Food insecurity						
Current	1.79 (1.26–2.55)	<1 (0.8)	1.56 (1.03–2.32)	11% (0.2)	2.42 (1.42–4.15)	<1% (0.9)
Persistent	1.79 (1.26–2.54)	<1 (0.4)	1.55 (1.04–2.29)	8% (0.3)	2.47 (1.44–4.25)	5% (0.1)

OR_ap_: pooled age-adjusted odds ratio.

Similar results were observed when the women with cognitive limitations were excluded from the analysis, although the proportion mediated through the education level was less notable.

There was no evidence of mediation through food insecurity (all p-values ≥ 0.4, Table 4). In contrast to the women without disabilities for whom the risk of sexual violence increased with the level of food insecurity, no association between the food insecurity level and sexual violence was found among the young women with disabilities. The ORs_ap_ for important versus no food insecurity were: 1.0, 95%CI 0.5–2.1 among women with disabilities and 2.5, 95%CI 1.3–5.0 among those without.

## Discussion

This pooled analysis of data collected from two large population-based surveys in sub-Saharan Africa provides additional evidence of the high vulnerability of women with disabilities to sexual violence [5,6]. Furthermore, it provides insights into the factors associated with this vulnerability, which could inform future interventions to prevent sexual violence against women with disabilities.

Our findings on the burden of sexual violence are in line with those of other studies and show that the risk of sexual violence among women with disabilities is twice that of women without disabilities [5,6]. The two studies were conducted in different contexts. Burundi has just emerged from a civil war, and a higher prevalence of violence was expected. Nevertheless, participants’ willingness to disclose their personal experience of (sexual) violence depends on the prevailing norms and other cultural factors (e.g. fear of retaliation), which could explain the observed difference. Interestingly, despite these differences, there was little heterogeneity in the ORs estimated across studies.

In contrast, we found substantial heterogeneity in the risk of sexual violence across the different types of limitations, which is important for future prevention efforts. Women with cognitive limitations had the highest risk of sexual violence and should be the focus of intensive preventive efforts. Although data on the vulnerability of women with cognitive disabilities living in low-income countries are limited, evidence from high-income countries indicates that this group is especially at risk of abuse [30]. In high-income countries, living in institutional settings, social isolation, (financial) dependence, and an absence of sexual education have been identified as risk factors for sexual abuse [31,32]. In this study, we found that a low but similar proportion of women with and without cognitive difficulties had discussions with their parents about sexuality. However, studies have shown that people with disabilities are often considered asexual, and their parents are more likely to be overprotective or reluctant to engage in discussions about sexuality with their growing children [33]. The content of the discussions may be different whether the child had disability or not. However, this topic was not explored in these studies.

Women with visual impairments were also found to be at high risk of sexual violence. This group has not received much attention thus far, and the limited data available come from high-income countries [34–36]. Therefore, there is an urgent need to confirm these findings and to better understand the risk factors associated with violence against women with visual impairments.

Beyond providing figures on the burden of sexual violence among women with disabilities in the African context, an important contribution of this analysis is its investigation into the factors associated with sexual violence. We found that women with disabilities experience multiple forms of disadvantage including poorer education, poorer housing, more frequent food insecurity and reduced social network. Our focus was to examine how these social and economic (dis)advantages shape the risk of sexual violence over women with disabilities’ life-course, and to what extent they contribute to women with disabilities’ vulnerability to sexual violence. Although women with disabilities were more likely to begin living with an intimate partner later than women without disabilities [12,37], we found that those who cohabitate early with an intimate partner were more vulnerable to sexual violence. It has been shown that sexual violence often occurs within the context of IPV [1]. Women with disabilities are less likely to choose their intimate partner, and several researchers have reported more frequent forced marriages among young people with disabilities, especially those with intellectual impairments [38–40]. Extreme cases include women with disabilities who were raped and then forced to marry the perpetrator [41,42]. In this quantitative research, such detailed information on the circumstances of cohabitation was not collected, and complementary qualitative research is therefore needed in order to better understand the issues.

Young women who began living with an intimate partner earlier in their life were more likely to have a low education level. Indeed, education level was found to be a strong mediating factor in the pathway between disability and violence and this suggests an important avenue for intervention. This result holds even after excluding women with cognitive limitations, for whom education level may be the clearest reflection of the severity of their impairment. Evidence on the relationship between education and violence is sometimes conflicting, as some studies have reported an increased risk of sexual violence among more educated women, while others have reported the opposite [42–44]. Different mechanisms have been proposed to explain the protective effect of higher education. First, girls and young women who are still studying live in more protective environments and are less likely to engage in early marriage or cohabitation [45]. Women with higher education levels are also more likely to challenge patriarchal norms that support violence against women and to find the resources to escape such violence when needed. However, the effect of this mechanism depends on contextual factors such as the prevailing norms. Higher levels of violence may occur in settings with strong patriarchal norms when these norms are challenged. Finally, more educated women can access greater economic opportunities later in life. In addition, in this study, the risk of sexual violence was not associated with food insecurity among women with disabilities, while it was twice as high among women without disabilities who experienced food insecurity, compared to those who did not experience food insecurity. This could result from the high prevalence of sexual violence and poverty observed in the group of women with disabilities.

Our study has several limitations. The main limitation is that community- and society-level risk factors were not fully captured. Social norms are an important factor that influences the risk of sexual violence and this was not quantitatively assessed in this study.

Another limitation concerns our ability to draw strong conclusions regarding causality and mediation due to the study design. Retrospective longitudinal data were collected to overcome the limitations of the cross-sectional design, but these data may be prone to recall bias. Although attention was given in the analysis to potential confounding factors, there may be residual unmeasured confounding factors responsible for bias. For instance, childhood living conditions were imperfectly captured through the reporting of food insecurity at age 10 years. Our analysis of the factors that could mediate the vulnerability of women with disabilities was only exploratory, and these results should be confirmed with interventions and prospective studies. Another possible limitation is related to the possible underreporting bias on sexual violence that is well described in the literature [46]. Nevertheless, to decrease this bias in the two studies, the interviewers received specific training on how to introduce this sensitive topic and how to provide support to any participants who expressed, or showed signs of, distress. In addition, the biographic interview helped to establish a more intimate relationship between the participants and interviewers, which, we believe, facilitated the disclosure of sexual violence. Another challenge encountered in this study was the evaluation of disability [47]. The pragmatic approach adopted in this study was to first focus on functional limitations using the WGSS questionnaire, which has been extensively evaluated and used [48]. However, notably, this instrument does not measure cognitive disability well, and this prompted us to add two additional questions to better capture this dimension.

Accordingly, this study shows the high burden of sexual violence among women and emphasises different factors that may increase the vulnerability of women with disabilities to sexual violence, specifically, early intimate partnership and a low education level. There is an urgent need for additional qualitative research to better understand the mechanisms at play in order to translate the research findings into preventive interventions.

## Supplementary Material

Supplemental Material

## Paper context

Available data from high-income countries indicates that women with disabilities have an increased risk of sexual violence, but little is known about the situation of those living in resource-limited countries. In this study, women with disabilities in Burundi and Cameroon were twice as likely to report sexual violence compared to women without a disability. Those with cognitive and/or visual limitation were the most vulnerable. Disability mainstreaming in prevention programmes that focus on violence against women is a priority for policy and action.
