# Supplementary material for: Burden of and risk factors for sexual violence among women with and without disabilities in two sub-Saharan African countries
Source: Glob Health Action. 2022 Jul 1;15(1):2077904. doi: 10.1080/16549716.2022.2077904 (PMC9255210; doi:10.1080/16549716.2022.2077904)
Supplement: Supplemental Material [file ZGHA_A_2077904_SM0655.docx]

**Washington Group questionnaire** To overcome the practical and conceptual difficulties in measuring disability, a group of experts set up by the UN Statistical Commission has proposed an operational tool for the identification of people with disabilities in surveys with good accuracy and reproducibility from one setting to another^1-3^. This tool includes a small number of questions covering six functional domains or basic actions: seeing, hearing, walking, cognition, self-care, and communication. Each question asks the respondent to rate on a four-point scale how much difficulty he/she has experienced in the domain (see below). The Washington Group questionnaire is available in various forms; a short set questionnaire includes six questions and is recommended for use in national survey because of its simplicity. Additional questions are available from the extended set to supplement those from the short set and provide more detail on functional limitations.

**Short set of the Washington Group questionnaire^1^**

Because of a physical, mental, or emotional health condition…

1. Do you have difficulty seeing even if wearing glasses?

2. Do you have difficulty hearing even if using hearing aid/s or are you deaf?

3. Do you have difficulty walking or climbing stairs?

4. Do you have difficulty remembering or concentrating?

5. Do you have difficulty (with self-care such as) washing all over or dressing?

6. Do you have difficulty communicating (for example, understanding or being

understood by others)?

**Two additional questions from the extended set of the Washington Group questionnaire were added in order to capture better people with intellectual disabilities:**

7. Do you have difficulty learning a new task, for example learning how to get to a new place?

8. Do you have difficulty analyzing and finding solutions to problems in day to day life?

Question response categories: No, Some, A lot, and Unable.

**Supplementary Figure**. Conceptual model


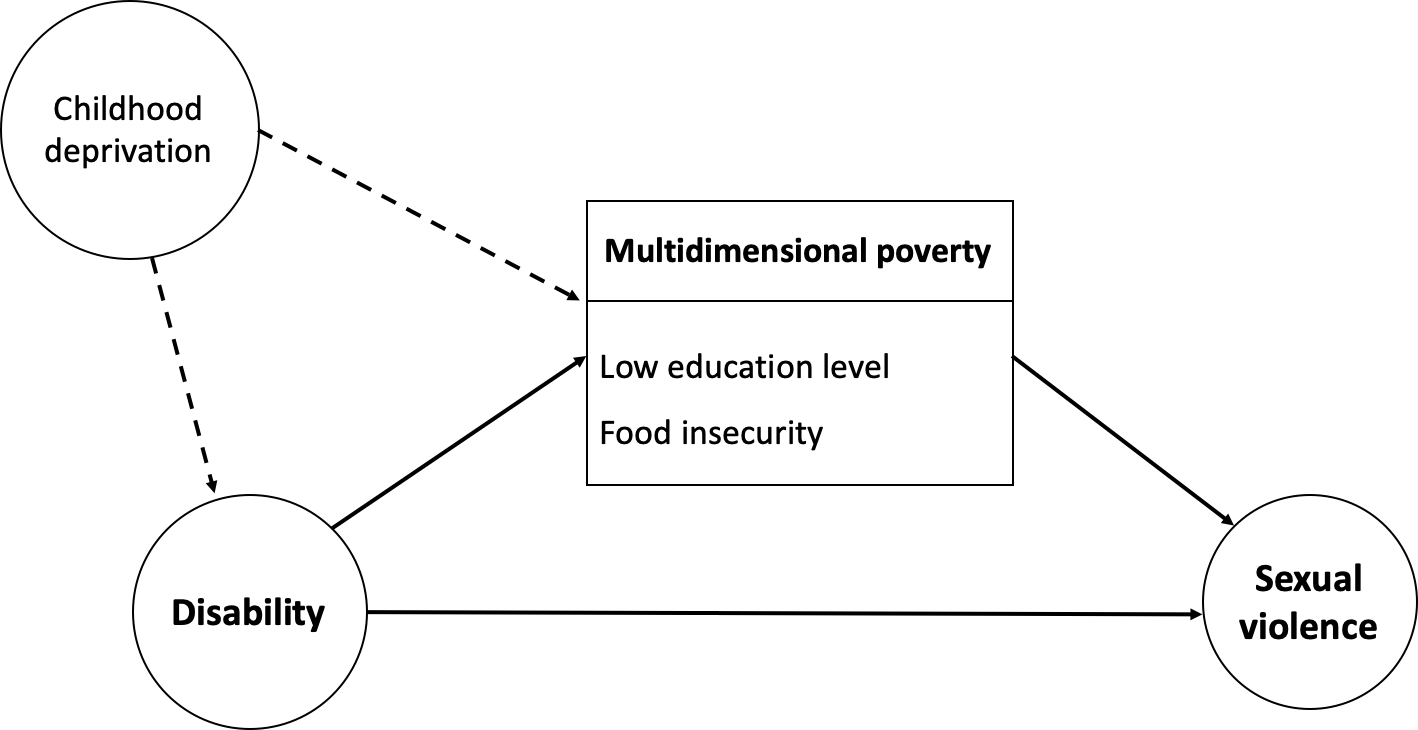


**Supplementary Table**. Description of the variables used in the analysis.

| **Label** | **Definition** | **Categories** |
| --- | --- | --- |
| Education level | Completed education level | - Primary not completed or lower level  - Primary (completed)  - Secondary level  - Higher education level |
| Persistent food insecurity | Food insecurity was assessed by asking the participants to rate their concern about getting food during the different periods of their life (from age 10 to the date of the survey). The levels of concern were assessed on a three-grade scale: 1 - no concern, 2 - some concern, 3 - important concern. | - Persistent food insecurity: participant reported important concern for all periods of the life-course history  (reference: any period with no or some concern only) |
| Food insecurity at age 10 | Level of concern at age 10 regarding access to food retrospectively reported by the participant. The level of concern is assessed on a three-grade scale: 1 - no concern, 2 - some concern, 3 - important concern. | Important concern versus no or some concern |
